# Evaluation of the new blood-pool CT contrast agent VivoVist in mouse models

**DOI:** 10.1371/journal.pone.0335025

**Published:** 2025-10-31

**Authors:** Eric W. Livingston, Jonathan E. Frank, Spencer V. Thompson, Gregory K. Wilkerson, Jean-Felix Presler, Hong Yuan

**Affiliations:** 1 Biomedical Research Imaging Center, The University of North Carolina at Chapel Hill, Chapel Hill, North Carolina, United States of America; 2 Department of Pathology and Laboratory Medicine, The University of North Carolina at Chapel Hill, Chapel Hill, North Carolina, United States of America; 3 Department of Radiology, The University of North Carolina at Chapel Hill, Chapel Hill, North Carolina, United States of America; Korea Institute of Radiological and Medical Sciences, KOREA, REPUBLIC OF

## Abstract

Small animal CT imaging provides high resolution imaging of bone structure, lungs, and gross anatomy. However, it is limited in its ability to provide high soft tissue contrast. Several blood pool CT contrast agents have been developed to enhance vascular and tissue contrast for preclinical imaging with varying enhancement capabilities. VivoVist^TM^ is the most recent commercially available blood pool CT contrast agent for preclinical applications. This study independently evaluated its radiopacity and tissue enhancement compared to two existing preclinical CT contrast agents, Mvivo-Au, and Fenestra-HDVC. Healthy nude mice were administered one of the three contrast agents. CT imaging was performed before and at 5 minutes, 1 hour, 4 hours, 24 hours, 48 hours, 96 hours, and 7 days post-injection. Tissue intensity and the enhancement ratio relative to pre-injection levels were quantified for each contrast agent at each time point. VivoVist demonstrated significantly higher blood enhancement compared to Mvivo-Au and Fenestra-HDVC at 5 minutes and 1 hour post-injection. However, the enhancement at 4 hours and later time points was inferior to that of Mvivo-Au. VivoVist exhibited the fastest blood clearance among the three contrast agents, with a blood half-life of 3.1 hours and was largely cleared from the blood by 24 hours post-injection. In CT imaging after 24 hours post-injection, VivoVist showed the highest liver enhancement, which remained high over the 7-day imaging period. Biodistribution assessment showed that the splenic uptake of VivoVist was extremely high. Histological examination of the tissues identified abundant contrast agent accumulation in the liver and spleen. No overt pathological changes were observed in either organ one month after the injection of VivoVist. Overall, the evaluation confirmed that VivoVist is an effective CT contrast agent for vascular and liver imaging with low toxicity. However, its relatively short blood half-life limits its use as a vascular contrast agent for a prolonged period.

## Introduction

X-ray computed tomography (CT) imaging has been widely utilized in preclinical animal models (referred to as micro-CT) to provide high resolution three-dimensional images of anatomical structures. In addition to its application in bone imaging, micro-CT imaging has been utilized with success to image lung and fat tissue in mouse models [1–4]. In vivo micro-CT imaging in live animals permits repeated imaging over time to monitor changes during experimental manipulation, providing an efficient and powerful tool for preclinical research with significantly reduced animal usage. One major limitation of CT is the inability to provide soft tissue contrast, which hinders the ability to differentiate vessels and other soft tissues. Thus, the use of contrast agents is frequently employed to enhance vascular or tissue contrast in CT imaging.

The most prevalent CT contrast agents are iodine-based non-ionic water-soluble compounds, such as iohexol, iodixanol, and iopromide. These agents have received FDA approval and are extensively utilized in clinical CT scans. However, these iodine-based clinical CT contrast agents are not optimal for small animal imaging due to their rapid clearance, which occurs within minutes [5]. This limitation is compounded by the fact that preclinical CT scanners are not typically equipped with high-speed gantries, which are necessary to facilitate rapid CT imaging before the clearance of the CT contrast agent. Thus, the scan time for animal CT imaging is longer, often spanning several minutes or more for high-resolution imaging, in contrast to the rapid imaging time observed in clinical CT scanners, which can complete a scan in seconds. As a result, blood-pool contrast agents, which can remain in blood vessels for extended periods, are particularly important in microCT imaging. These agents facilitate the delineation of vascular networks and surrounding tissue through contrast enhancement.

A considerable number of blood-pool contrast agents have been subjects of research and development. The earliest development of blood-pool contrast agents was based on diatrizoate-carrying liposomes, which was reported in 1984 [6,7]. Subsequently, a number of contrast agents have emerged in the following decades. These agents are mostly based on nanoparticles, including lipid emulsion particles [8,9], polymer micelles [10], and pegylated nanoparticles [11] among others. The radiopaque elements also extend from iodine to other elements with a high atomic number, such as barium [12], gold [13], and bismuth [14]. Fenestra™ (alpha-Tocopheryl 2,3,5-triiodobenzoate) is the first commercially available blood-pool contrast agent for preclinical CT imaging. It is an iodine-based lipid emulsion. The early version of Fenestra contained 50 milligrams of iodine per milliliter, but a newer version, designated as Fenestra-HDVC (MediLumine Inc, Montreal, QC, Canada), exhibits the capacity to load a two-fold higher amount of iodinated material, achieving 100 mg of iodine per milliliter. Several gold-based nanoparticles are also commercially available for vascular imaging, including AuroVist™-15nm (Nanoprobes Inc, Yaphank, NY USA) and Mvivo-Au (MediLumine Inc, Montreal, QC, Canada). AuroVist-15nm is provided at 200 mg/ml concentration of gold in the form of biocompatible 15 nm gold nanoparticles [15]. Mvivo-Au features core particles measuring approximately 15 nm and is available at a concentration of 200 mg/ml of gold [16]. VivoVist^TM^ (Nanoprobes Inc, Yaphank, NY USA) is the most recent commercially available blood pool CT contrast agent (available in 2024) for preclinical applications. It is an alkaline earth metal-containing nanoparticle with an average particle size of approximately 150 nm. To date, there has been no independent evaluation of this novel blood-pool contrast agent for small animal imaging.

In this study, we evaluated the new contrast agent, VivoVist, by comparing its vascular enhancement, liver enhancement, and blood clearance with two other blood-pool contrast agents: Fenestra-HDVC, an iodine-based contrast agent, and Mvivo-Au, a gold-based contrast agent. The tissue toxicity of VivoVist was also evaluated by examining the liver, kidney, and spleen tissue at one month after injection.

## Materials and methods

### Animals

A total of eleven healthy athymic mice (NCr-nu/nu inbred mice, ~ 6–10 weeks old, mixed male and female) were used for the CT contrast agent evaluation. Ten of the mice were assigned to one of the three contrast agents: VivoVist (n = 3), Mvivo-Au (n = 4), or Fenestra-HDVC (n = 3). An additional healthy mouse was used as a control without any contrast agent injection or imaging for histology assessment comparison. Detailed imaging procedures and dosing information are described in the *in vivo* CT imaging section below. All mice were monitored daily during the seven-day imaging period for any signs of pain or distress, with weekly checks continuing thereafter. Animals that showed 20% or more weight loss or a decrease in body condition score from 3 to 2 were to be immediately euthanized. All mice were euthanized 30 days after the contrast agent injection via anesthesia overdose followed by cervical dislocation, and organs of some mice were collected for histological analysis.

All animal procedures were performed in strict compliance with ethical regulations for animal research and were approved by the Institutional Animal Care and Use Committee (IACUC) of the University of North Carolina at Chapel Hill. Considerations were given in the study design to minimize or avoid discomfort, distress, and pain, and reduce the number of animals by using repeated imaging in the same animal. All animal handlers were well-trained and certified to perform related procedures on animals.

### *In vivo* CT imaging

Live animal CT imaging was conducted in the Preclinical Molecular Imaging Facility at the University of North Carolina at Chapel Hill. A high-resolution animal CT system (Quantum GX2, Revvity Inc, Waltham MA) was used to obtain x-ray CT images. The system was calibrated for Hounsfield units (HU) using the vendor-provided QC phantom before use.

Before imaging, the mice were anesthetized using a 3% isoflurane/oxygen mixture for induction and maintained at 2% isoflurane for all subsequent imaging procedures. A catheter with a 30 G needle was placed in the mouse tail vein for administration of the contrast agents. Subsequently, the mice were placed on a heated imaging cradle with temperature maintained at 37 °C. Baseline CT imaging was first performed prior to contrast injection. A multi-bed low-dose CT imaging protocol was used. Images were acquired using a fixed filter comprised of 0.5 mm aluminum and 0.06 mm copper with the following parameters: 90 kV peak voltage, 88 µA current, 72 mm acquisition field of view (FOV), standard scan mode with a scan time of 2 minutes per bed, and total imaging time of 6 min per mouse. Projection images were reconstructed using the proprietary reconstruction method with a 60 mm FOV and an isotropic voxel size of 120 microns. The radiation dose from each acquisition was approximately 100 mGy on exposed tissue.

The contrast agent was injected as a bolus dose through the tail vein catheter after the baseline CT scan without moving the animal. The injection dose for both VivoVist and Mvivo-Au was 1 g/kg based on the radiopaque element for direct comparison. The injection dose for Fenestra-HDVC was 0.5 g/kg due to the allowed intravenous injection volume limit. The 1 g/kg dose was recommended by the VivoVist manufacturer. Based on the concentration of the radiopaque particles in the stock solutions, the volume doses for the 1 g/kg mass dose were 3.3 ml/kg, 5 ml/kg, and 10 ml/kg for VivoVist, Mvivo-Au, and Fenestra-HDVC, respectively. According to our IACUC guidelines, the maximum allowable volume dose for intravenous injections is 5 ml/kg in mice. Thus, the volume dose for the Fenestra-HDVC group was reduced to 5 ml/kg. Resultantly, the dose for the Fenestra-HDVC is 0.5 g/kg, instead of 1g/kg. The detailed dosage information, as well as other related contrast agent information, is presented in Table 1.

**Table 1:** General characteristics of the three blood pool contrast agents and dose.

Agent	Radiopaque Element	Stock Concentration ^c^	Particle Size	Injected Dose	Injected volume
**VivoVist™**	Barium ^a^	300 mg/ml	150 nm	1 g/kg	3.3 ml/kg
**Mvivo™-Au**	Gold	200 mg/ml	15 nm	1 g/kg	5 ml/kg
**Fenestra™-HDVC**	Iodine	100 mg/ml	84 nm	0.5 g/kg ^b^	5 ml/kg

^a^. As determined by Badea et al [17], not provided by the manufacturer;

^b^. The maximum allowed mass dose based on the maximum allowed volume dose for intravenous injection in mice;

^c^. The concentration of the radio-opaque element in stock solution.

CT imaging was conducted at 5 min and 1 hour following the injection of the contrast agents using the same imaging protocol as the baseline scan. Mice were allowed to recover from anesthesia after the 1 hour imaging time point. They were re-anesthetized using isoflurane inhalation, and repeated scans were performed at 4 hours, 24 hours, 48 hours, 96 hours, and 7 days post-injection using the same imaging protocol.

After the completion of the imaging study at seven days post-injection, the animals were observed for an additional three weeks for any signs of adverse effects. All mice were euthanized 30 days after the contrast injection. For the mice injected with VivoVist, the liver, kidney, and spleen were collected for histological analysis. The control mouse that did not receive any contrast agent injection was euthanized as well, and its organs were collected for histological comparison.

### Image analysis

CT Images were processed and analyzed using 3D Slicer (https://www.slicer.org/). In each animal at each timepoint, the tissue intensity in Hounsfield units (HU) was measured in the left ventricle, liver, kidney, spleen, cerebrum, and forelimb muscle using representative regions of interest. These tissues were chosen to provide a comprehensive representation of the biodistribution. To measure the tissue uptake in each organ, an ellipsoid volume of interest (VOI) was placed on the selected organ with the size adjusted for the best representation. The VOIs were saved and applied to the next image sets with minimal adjustment. Thus, the volume of VOIs was kept consistent across all animals and timepoints, and each was located at similar positions within each animal. The left ventricle intensity served as an analog for the blood pool intensity, and the time course of blood and tissue intensity was measured up to 7 days post-injection of each contrast agent. The enhancement ratio was defined as the measured intensity of a tissue at each timepoint divided by the baseline intensity (before contrast agent injection) from the same animal. The mean intensities of each organ were measured for each agent at each timepoint for biodistribution assessment.

To estimate the blood half-life of each contrast agent, the blood kinetic curves were analyzed using exponential modeling. First, the baseline signal before the contrast injection was subtracted from each blood intensity kinetic curve. Then, the kinetic curves were fitted to a one-phase exponential model through non-linear fitting using GraphPad Prism (version 10.4.1). The model equation can be described as: *I(t)*=*I*_0_*e*^*–kt*^, where *I(t)* is the blood intensity at a given time, *I*_0_ is the initial blood intensity right after bolus injection, and k is the decay time constant. Once k is estimated from fitting the exponential model, the blood half-life (T_1/2_) can be calculated as T_1/2_ = ln(2)/k.

### Histology

Three mice with VivoVist injections and one control mouse were euthanized at 30 days following the contrast injection, and the liver, kidney, and spleen were collected. Tissues were fixed in 10% neutral-buffered formalin for one week and embedded in paraffin for sectioning. Tissue sections (5 µm thick) of each organ were stained with hematoxylin and eosin stain using standardized protocols at the University of North Carolina Animal Pathology Core facility and evaluated by an experienced veterinary pathologist.

### Statistics

Statistical analysis was performed using GraphPad Prism (Version 10.4.1). All results are expressed as mean  ± standard deviation. Based on prior knowledge of the normal distribution of contrast-enhanced CT signal intensity measurements, one-way ANOVA with post-hoc Tukey’s test for multiple comparisons was selected to compare the signal intensity among three CT contrast agents, as opposed to non-parametric analysis. Statistically significant differences are indicated when p < 0.05.

## Results

### Vascular enhancement and blood half-life

**Fig 1:**
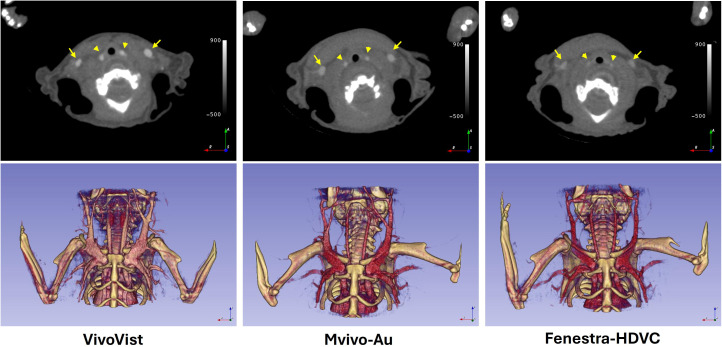
Contrast enhanced vascular images and volume rendering of CT images in mice at 5 min post the injection of the contrast agents. Major vessels, including the carotid artery (arrow heads) and the jugular vein (arrows) are clearly visible in contrast-enhanced CT images. Smaller vessels around the major vessels can be also segmented as shown in the volume rendering images. VivoVist showed the strongest enhancement right after the injection of the contrast agents.

In vivo CT images were obtained before and at 5 min, 1 hour, 4 hours, 24 hours, 48 hours, 96 hours, and 7 days following the injection of VivoVist and the other two contrast agents, Mvivo-Au and Fenestra-HDVC, for comparative analysis. There were no significant differences in animal body weights among the three contrast agent groups. The contrast agent was given per body weight of individual animal. The skin color of the mice injected with Mvivo-Au turned dark blue likely due to the dark color of the gold nanoparticles. No other adverse effects or abnormal behavior were observed in all mice after the injection of the contrast agents. No animals reached humane endpoints or died unexpectedly before meeting them. The measurements of blood signal intensity demonstrated a significant enhancement of vasculature in response to all three contrast agents, as illustrated in Fig 1. The blood pool intensity from VivoVist was significantly higher than that from Mvivo-Au or Fenestra at the 5 min and 1-hour time points but not at the 4-hour time point and beyond. At 24 hours post-injection, the blood pool intensity from Mvivo-Au was significantly higher than that from VivoVist and Fenestra. The vascular enhancement ratio from VivoVist reached 12, the highest among the three contrast agents, at 5 minutes post-injection and quickly dropped to 5.7 at 4 hours post-injection, a level similar to the enhancement from Fenestra-HDVC. The enhancement from Mvivo-Au remained relatively stable, changing from 8.3 at 5 min to 7.3 at 4 hours post injection. At 24 hours post-injection, the enhancement ratio for VivoVist was close to 1, indicating little enhancement at this time point. The mean blood intensity and mean vascular enhancement ratio from each contrast agent are shown in Table 2. Detailed data of blood and tissue intensity measurements are available in the Supporting Information (Table A & Table B in S1 file). Please note that the Fenestra-HDVC dose was half that of the other two contrast agents. This was due to the permitted bolus injection volume being limited in mouse studies.

**Table 2:** Blood half-life, mean blood pool intensity, and mean vascular enhancement.

Agent	Half-life (hour)	Blood Pool Intensity (HU) (mean ± SD)				Mean Enhancement Ratio			
		5 min ^^a^^	1 h ^b^	4 h ^^c^^	24 h ^d^	5 min	1 h	4 h	24 h
**VivoVist (n = 3)**	3.1 ± 0.5	439.1 ± 49.8	348.2 ± 31.0	208.7 ± 31.2	40.5 ± 2.7	12.0	9.4	5.7	1.1
**Mvivo-Au (n = 4)**	28.5 ± 1.8	281.4 ± 45.2	260.8 ± 41.2	247.8 ± 46.3	170.7 ± 20.0	8.3	7.7	7.3	5.1
**Fenestra-HDVC (n = 3)**	9.9 ± 1.3	238.5 ± 9.8	232.4 ± 11.4	217.1 ± 13.0	65.6 ± 16.5	6.0	5.9	5.5	1.6

^a^, VivoVist group was significantly higher than the other two groups, p < 0.005

^b^, VivoVist group was significantly higher than the other two groups, p < 0.005

^c^, No significant difference between any two groups

^d^, Mvivo-Au group was significantly higher than the other two groups, p < 0.0001

**Fig 2:**
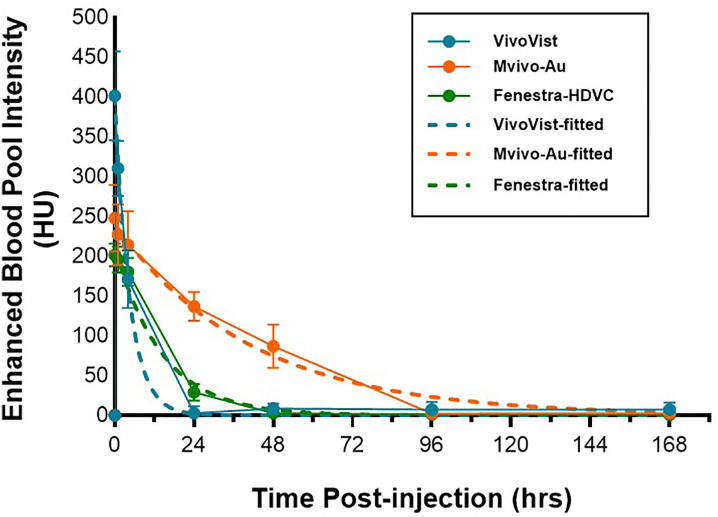
Blood kinetics of three blood-pool contrast agents. Baseline subtracted CT enhancement signal was plotted over time up to 7 days post-injection. The blood signal intensity curves were fitted with a one-phase exponential decay model to estimate the blood half-life.

The time course of vascular intensity was plotted for all three contrast agents up to seven days post-injection (Fig 2). The baseline-subtracted blood curves were modeled to a one-phase exponential decay equation to estimate the blood half-life. The R-squared values for all non-linear fittings reached over 0.98, indicating the goodness of fit with this model. As shown in Table 2, Mvivo-Au exhibited the longest blood half-life (28.5 hours, ranging from 26.4 to 30.6 hours), while VivoVist had the shortest half-life (3.1 hours, ranging from 2.7 to 3.7 hours)**.** Most contrast signals were cleared out by 24 hours post-injection of VivoVist and Fenestra-HDVC, limiting their application for vascular imaging on the second day after contrast injection. Mvivo-Au showed the longest blood retention, with the contrast still detectable at 2 days post-injection.

### Biodistribution and enhancement in liver and spleen

**Fig 3:**
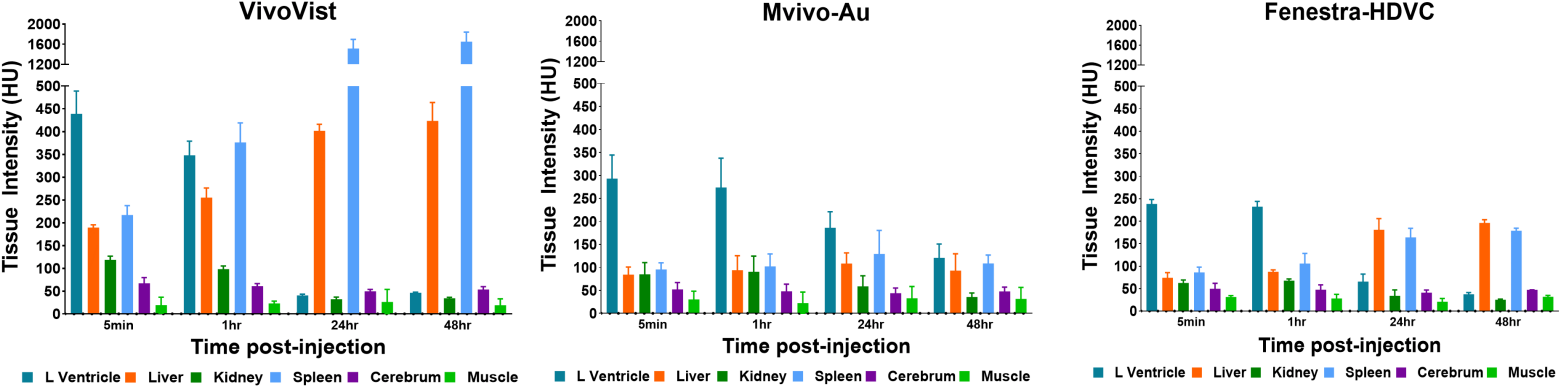
Biodistribution of the three contrast agents in mice. CT signal in left ventricle, liver, kidney, spleen, cerebrum and muscle was measured at various time points after injection. VivoVist showed much higher blood enhancement compared to the other two contrast agents at early time points. The distribution was comparable between Mvivo-Au and Fenestra-HDVC. VivoVist accumulated primarily in the liver and spleen after 24 hours, reaching very high accumulation level in the latter.

The biodistribution of the contrast agents was measured by quantifying CT intensity in various organs at different times post-injection. As illustrated in Fig 3, a notable uptake was observed in the liver and spleen following the injection of VivoVist. After 24 hours, VivoVist had accumulated primarily in the liver and spleen. A comparable biodistribution was observed for the Mvivo-Au and Fenestra-HDVC contrast agents with liver and spleen being the primary clearance organs. However, the VivoVist agent exhibited an exceptionally high uptake in the spleen compared to the other two contrast agents.

**Fig 4:**
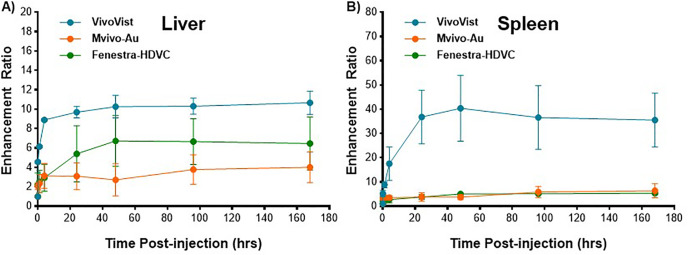
Time course of the enhancement in liver and spleen after the injection of VivoVist, Mvivo-Au, and Fenestra-HDVC. The enhancement ratio was the ratio of the measured tissue intensity at each time point to the baseline intensity before the injection of the contrast agent from the same animal. VivoVist provided the highest enhancement in liver and spleen compared to the other two contrast agents, and the enhancement remained high throughout the imaging study. In addition, the uptake of VivoVist in the liver was much faster than Fenestra-HDVC, with a steeper slope. VivoVist also produced significantly higher enhancement in the spleen than Mvivo-Au and Fenestra-HDVC.

**Fig 5:**
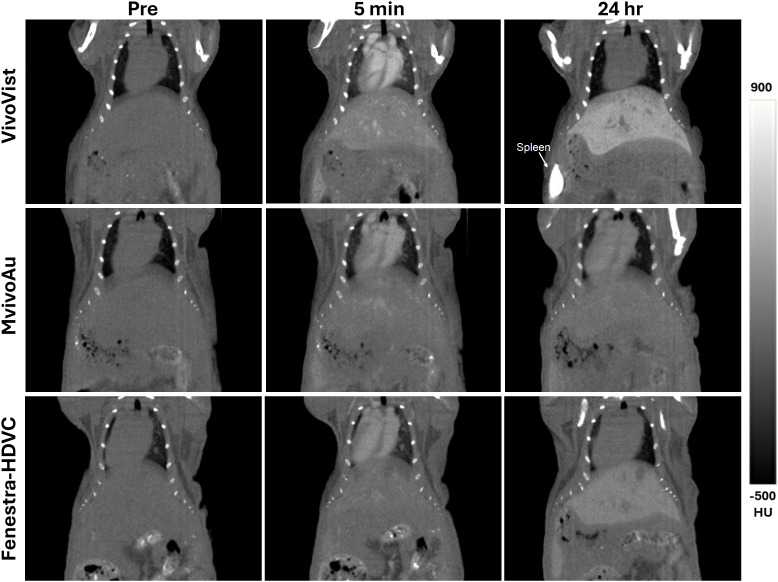
CT images of mice after the injection of VivoVist, Mvivo-Au, and Fenestra-HDVC. Images taken at baseline before the injection, right after injection, and one day after injection are shown. Enhancement in vasculature (including heart ventricles), liver, and spleen can be seen at different time points for each tracer. VivoVist showed the highest vascular enhancement right after injection, and produced the highest liver and spleen enhancement by the 24-hour timepoint.

The time courses of the uptake in the liver and spleen are illustrated in Fig 4. The spleen signal was over 40 times higher than the baseline signal at 48 hours post-injection and remained high at one week post injection (Fig 4a). Liver enhancement was compared among the three contrast agents. VivoVist demonstrated the most pronounced liver signal among the three agents (Fig 4b). Note that the liver signal at 4 hours post-injection contains both signals in the vasculature and in the liver tissue; thus, liver enhancement is mainly analyzed in images at 24 hours post-injection and beyond. The CT signal in liver at 24 hours post-injection was significantly higher (p < 0.001) in VivoVist-injected mice than in Fenestra-HDVC-injected mice. It is noteworthy that the Fenestra-HDVC dose was half of the mass dose of VivoVist due to volume limits. As demonstrated in Fig 5, VivoVist, similar to Fenestra-HDVC, possesses the capability of two-phase imaging. In the early phase, it functions as a blood-pool contrast agent to delineate vasculature, and in the late phase at 24 hours or later, it functions as a liver enhancer to demarcate the liver. In contrast, the Mvivo-Au agent primarily functions as a blood-pool contrast agent and exhibits significant enhancement of the vasculature in the days following injection.

### Histopathology findings

**Fig 6:**
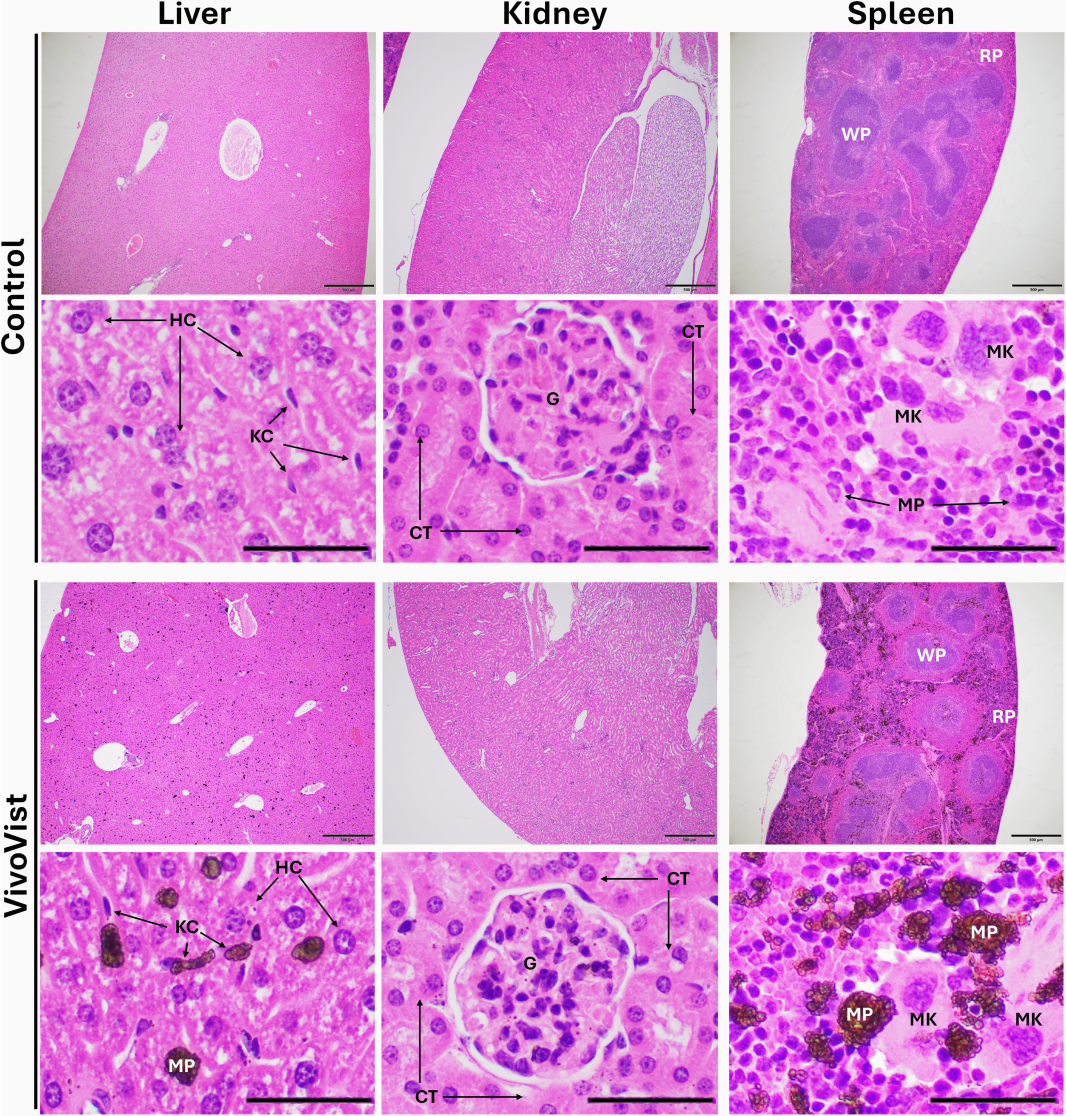
Histology examination of tissues from a healthy control mouse and a mouse at 1 month after the injection of VivoVist. Liver, kidney, and spleen sections after H&E staining are shown in low (4x) magnification (500 µm/bar) and high (20x) magnification (100 µm/bar). There were significantly high accumulations of pigment (presumed VivoVist particles) in both the liver and spleen. Splenic 20x images are of areas of extramedullary hematopoiesis. Liver: HC = hepatocyte; KC = Kupffer cell; MP = macrophage (presumed). Kidney: G = glomerulus; CT = cortical tubular cells. Spleen: RP = red pulp; WP = white pulp with lymphoid follicles; MK = megakaryocytes, MP = macrophage (presumed). No overt pathologic changes were identified in any of the three organs at one month after the injection of VivoVist at the dose of 1 g/kg.

Tissues from one age-matched, untreated (control) mouse and three mice injected with VivoVist one month prior to collection were examined microscopically. Fig 6 shows H&E staining of the liver, kidney, and spleen collected from the control and one VivoVist injected mouse. Histological findings for the tissues were consistent in all three VivoVist injected animals. In the liver of both the control and VivoVist injected animals, there were mild diffuse vacuolar changes consistent with glycogen accumulation and occasional areas of mild lymphoplasmacytic perivascular inflammation of the central vein and portal areas. In the VivoVist injected animals alone, there were moderate dark brown pigment accumulations scattered throughout the liver parenchyma. These accumulations are presumed to be VivoVist contrast agent nanoparticles. Based on the size, shape, and location of the accumulations, the contrast agent appeared to primarily be contained within Kupffer cells and macrophages, with smaller quantities of pigment associated with areas of extramedullary hematopoiesis, and the vascular stroma. Endothelial cells and hepatocytes occasionally had small numbers of intracytoplasmic pigment granules as well. Other than the pigment accumulation, there were no overt pathologic changes noted in the liver.

In the kidney tissue, there were small numbers of individual dark brown pigment granules (presumed VivoVist nanoparticles) located within the cells of the glomerulus, tubules, and free within the interstitial space of the cortex. Pigment granules were also sometimes seen in the medullary tubular cells but were much less common than in the cortical tubular cells. There were no overt pathologic changes identified in the kidneys and pigment accumulation was the only consistent histologic finding.

In the spleen, there were marked accumulations of dark brown pigment (presumed VivoVist nanoparticles) in the red pulp and occasional smaller accumulations in the germinal center of lymphoid follicles (white pulp). These accumulations appeared to primarily be contained within macrophages although some granular accumulations were also identified outside of the cells, free within the stroma of the splenic parenchyma. Many of the densest pigment accumulations in the red pulp were centered on areas of extramedullary hematopoiesis. Notably, hematopoiesis appeared similar between the control and contrast injected animals. Other than pigment accumulations, there were no overt pathologic changes identified in the spleen.

## Discussion & Conclusion

The present study evaluated the newly developed CT contrast agent, VivoVist, in terms of its vasculature enhancement, blood half-life, biodistribution, and liver enhancement in comparison with two other commercially available contrast agents, Mvivo-Au and Fenestra-HDVC, in the athymic mouse model. The findings revealed that VivoVist exhibited a notable capacity for vascular enhancement, demonstrating the highest enhancement ratio within the initial time (0–4 hours) post-injection among the three contrast agents. However, its blood half-life and blood retention were found to be the least compared to the other two contrast agents, and it was mostly cleared out at 24 hours post-injection, making it impossible for imaging vasculature the second day after injection. Furthermore, VivoVist demonstrated superior liver enhancement in comparison to the other two contrast agents, showing potential as a contrast agent for liver imaging. The highest accumulation of VivoVist was observed in the spleen. Histological evaluation revealed that VivoVist mainly accumulated in the liver’s Kupffer cells and macrophages rather than in hepatocytes. This indicates that VivoVist enters the liver via passive penetration rather than active interaction with hepatocytes, unlike Fenestra-HDVC. The significant accumulation in the spleen was primarily observed in macrophages within the red pulp, suggesting that splenic filtration is a primary clearance route. No overt pathological changes were identified in either the liver or spleen, where the VivoVist agent accumulated predominantly. These histological findings suggest that VivoVist is a low-toxicity contrast agent.

VivoVist is a recently developed, commercially available blood-pool contrast agent. The manufacturer has not disclosed the radiopaque element in the product note or on their website but stated it as an alkaline earth metal. Based on the spectral decomposition analysis work from Badea et al, VivoVist is a barium-based CT contrast agent (17). In their collaboration with the manufacturer, Badea et al. reported the application of their photon-counting microCT imaging in combination with the VivoVist contrast agent to enhance vascular imaging, and confirmed the barium content in VivoVist through the spectral decomposition analysis. We conducted an independent evaluation to provide unbiased assessment regarding its contrast enhancement capacity in general mouse imaging. The data obtained from this evaluation confirmed the agent’s significant potential for vascular enhancement and revealed its sub-optimal blood retention. The blood half-life for VivoVist is reported to be 14 hours on the manufacturer’s website; however, our in vivo imaging data consistently determined a blood half-life of ~3 hours. The discrepancy on the half-life estimation could be due to using different PK models. In our study, the blood half-life was determined by the time constant from the one-phase exponential fitting (see the method section). The one-phase exponential model, or one-compartment PK model, has been commonly used to determine drug half-life [18, 19]. Some essential criteria to use the one-phase exponential model include that the drug distribution space is mainly in the plasma and that the elimination occurs directly from the blood without reabsorption [19]. We have compared the one-phase and two-phase exponential models for fitting, and one-phase exponential fitting was chosen due to its better Goodness of Fit evaluated via mean squared error. Even using two-phase exponential PK model, the estimated blood half-life for the slow phase of VivoVist was less than 4 hours (data not shown). However, it is unclear how the 14 hours of blood half-life was determined by the manufacture as there were no references on the vendor website. A number of studies have reported the evaluation and application of Mvivo-Au [16, 20] and Fenestra-HDVC [21–23]. The findings of the present study are consistent with those of previous reports on Mvivo-Au and Fenestra-HDVC. The long-lasting Mvivo-Au contrast agent is useful for monitoring changes in vasculature over multiple days. Based on our assessment, repeated injections will be needed for both VivoVist and Fenestra-HDVC in order to monitor changes of vasculature over the course of several days.

VivoVist also showed great potential for liver imaging. Enhancement from VivoVist in liver was significantly higher than that of Fenestra-HDVC. The uptake of Fenestra-HDVC in liver is mainly driven by its hepatocyte-selective apolipoprotein E (ApoE) ligands on its surface, leading to selective binding to ApoE receptors in healthy hepatocytes [21, 23]. The uptake and retention mechanism of nanoparticles in liver highly depends on their size, shape, surface charges, and surface ligands. Without knowing some key information, the exact liver uptake mechanism for VivoVist is unclear. Based on the dynamic uptake curves and histological examination, we speculate that its liver uptake involves mainly passive vascular extravasation without selective binding or interaction with hepatocytes. Among the dynamic tissue uptake curves of the three contrast agents (Fig 4), VivoVist showed a fast liver enhancement in the first 5 min ~ 1 hour post injection and stayed stable after 4 hours post injection, suggesting a direct passive penetration through porous vasculature. Fenestra-HDVC takes much longer to reach a plateau and showed a reduction trend after three days. Based on our histological analysis, the nanoparticles accumulate throughout the liver parenchyma but are primarily contained within Kupffer cells and macrophages, with little accumulation in hepatocytes, further confirming its low interaction with hepatocytes. Fenestra-VC is an early blood-pool CT contrast agent with both vascular enhancement and liver imaging capabilities [23, 24]. Compared to Fenestra-VC, Fenestra-HDVC contains double the iodine concentration (100 mg/ml) and exhibits a higher enhancement power compared to its earlier version. In comparison to Fenestra-HDVC, VivoVist demonstrated superior liver enhancement when an equivalent volume of contrast agent was injected, indicating its considerable liver imaging potential, despite its non-specific hepatocyte uptake. There are several other CT contrast agents that can be used for liver imaging, including Exitron nano 6000 and Exitron nano 12000 [25, 26]. Boll et al conducted detailed evaluation between them and Fenestra-LC, and showed that Exitron contrast agents provided stronger contrast enhancement in liver with lower injection volume. Nota also showed high potential for Exitron nano12000 for liver imaging in rats [26]. However, the enhancement ratio for the Exitron agents was less than 2 [22], while the enhancement ratio for VivoVist reached as high as 10 in the liver (Fig 4). Based on this finding, VivoVist could be useful for quantifying liver volume and delineating surrounding abdominal structures.

One concern about VivoVist was its extremely high uptake in the spleen. The signal intensity in the spleen was about 40 times greater than the baseline level after the injection of VivoVist, and the high accumulation of the VivoVist particles can remain for a substantially long time (at least a month in our assessment). This prompted us to evaluate any potential toxic effects of VivoVist on the spleen. The histological examination revealed no pathological changes in the liver, kidney, or spleen after the injection of a dose at 1g/kg dose. According to the manufacturer’s product information, the maximum tolerated dose for VivoVist is > 4 g/kg. Given the prolonged, slow clearance rate in the spleen, we recommend that the total accumulated dose be recorded in multi-dose injection studies for each animal.

The quality of image contrast when employing contrast agents also depends on the imaging protocol, including the x-ray energy level, imaging time, and whether gating is used. The K-edge of barium, referring to the abrupt increase in photoelectric absorption of x-ray photons, is 37.4 keV [17]. This is similar to iodine, which has a K-edge energy of 33.2 keV. For barium, this indicates that an x-ray with an energy level of approximately 37 keV should provide the highest absorption, resulting in the optimal image contrast [27]. However, it has been shown that x-ray CT at 37 keV energy can result in high noise levels using common commercial preclinical CT systems. This can be attributed to the significant tissue absorption and the attenuation of low-energy x-rays. We employed an imaging protocol that utilized 90-keV x-ray energy, which enabled the acquisition of high-quality images with high tissue penetration and high signal-to-noise ratio (SNR). This energy level might be more beneficial for imaging gold nanoparticles, as the K-edge energy for gold is 80.7 keV. The acquisition protocol that we have used is a fast two-minute imaging protocol excluding the gating option. This was designed mainly with an important consideration on the accumulated radiation dose in repeated CT scan studies. The CT protocol in this study produces approximately 100 mGy of radiation per scan, which is considered a low-dose radiation protocol. This allows for repeated imaging over time on the same animal without excessively high radiation exposure. The enhancement measured with this protocol would be more practical for longitudinal imaging studies with blood-pool contrast agents. Gated imaging protocols can be beneficial when assessing heart, lung, and liver tissue because the motion can negatively affect the accuracy of the size and small structures [28, 29].

This study was not without limitations. Only two established CT contrast agents with different compositions were chosen for comparison with VivoVist. Other currently available agents may offer comparable or better results in different applications. Given that VivoVist has relatively shorter blood half-life, it would be more accurate to determine the blood half-life if we could take more imaging time points between 4 hours and 24 hours. For the tissue toxicity evaluations, the results were determined from histological analysis of H&E-stained tissue at 30 days post injection. It would be ideal to collect blood samples for assessment to have more complete information on systemic toxicity.

In summary, the study provided an independent evaluation of the blood-pool contrast agent VivoVist for *in vivo* animal imaging. Our results confirmed its effectiveness in imaging vasculature and its potential for liver imaging. However, its blood half-life is relatively short, limiting its use as a vascular contrast agent over a prolonged period.

## Supporting information

S1 File Detailed data of CT signal intensity in blood and tissue. (DOCX)
