# Supplementary material for: Evaluation of the new blood-pool CT contrast agent VivoVist in mouse models
Source: PLoS One. 2025 Oct 31;20(10):e0335025. doi: 10.1371/journal.pone.0335025 (PMC12578146; doi:10.1371/journal.pone.0335025)
Supplement: S1 File — (DOCX) [file pone.0335025.s001.docx]

**Supporting Information**

**Evaluation of the new blood-pool CT contrast agent VivoVist^TM^ in mouse models**

Eric W. Livingston ^1^, Jonathan E. Frank ^1^, Spencer V. Thompson ^1^, Gregory K. Wilkerson ^2^, Jean-Felix Presler^1^, Hong Yuan ^1, 3 *^

^1^ Biomedical Research Imaging Center, The University of North Carolina at Chapel Hill, Chapel Hill, North Carolina, USA; ^2^ Department of Pathology and Laboratory Medicine, The University of North Carolina at Chapel Hill, Chapel Hill, North Carolina, USA;^3^ Department of Radiology, The University of North Carolina at Chapel Hill, Chapel Hill, North Carolina, USA

**Detailed data of CT signal intensity**

The study independently evaluated a new CT contrast agent, VivoVist^TM^ regarding its blood and tissue enhancement by comparing it with two existing preclinical CT contrast agents, Mvivo-Au, and Fenestra-HDVC. The contrast agent was injected as a bolus dose into healthy mouse models through the tail vein catheter after the baseline CT scan. Signal intensity in the blood (as measured in the left ventricle) and in key organs was measured at a series of time points.

Detailed signal intensity measurements in blood were presented in **Table A**. The CT signal intensity in key organs and muscle from each contrast agent was presented in **Table B**.

**Tabel A.** **Blood signal changes over time**. Blood signal intensity (Hounsfield Unit (HU)) was measured from the left ventricle of individual animals before and at various time points post injection. The dose of the contrast agent was 1 g/kg (radiopaque elements) for VivoVist and Mvivo-AU, and 0.5 g/kg for Fenestra-HDVC. The Fenestra-HDVC dose was limited by the permitted volume for intravenous injection.

| Agent | **VivoVist** | | | **Mvivo-Au** | | | | **Fenestra-HDVC** | | |
| --- | --- | --- | --- | --- | --- | --- | --- | --- | --- | --- |
| Animal | **M1** | **M2** | **M3** | **M4** | **M5** | **M6** | **M7** | **M8** | **M9** | **M10** |
| **Pre-Inj** | 30.9 | 43.3 | 39.3 | 35.4 | 31.1 | 39.8 | 29.3 | 43.8 | 33.3 | 43.8 |
| **5min** | 492.9 | 394.5 | 429.9 | 284.3 | 306.6 | 317.8 | 216.9 | 234.2 | 249.6 | 231.5 |
| **1hr** | 355.8 | 314.1 | 374.6 | 276.8 | 281.5 | 285.5 | 199.3 | 229.1 | 245.2 | 223.0 |
| **4hr** | 227.0 | 172.6 | 226.4 | 269.3 | 256.5 | 284.9 | 180.6 | 231.5 | 212.9 | 206.7 |
| **24hr** | 42.6 | 37.5 | 41.5 | 182.0 | 183.0 | 176.7 | 140.9 | 57.7 | 54.5 | 84.6 |
| **48hr** | 46.6 | 47.6 | 44.9 | 124.9 | 135.0 | 145.6 | 76.8 | 40.2 | 34.7 | N/A |
| **96hr** | 49.2 | 45.8 | 40.7 | 37.6 | 43.4 | 37.1 | 27.5 | 34.9 | 34.8 | 48.5 |
| **7Days** | 48.1 | 45.0 | 41.9 | 47.1 | 33.9 | 33.0 | 33.0 | 35.1 | 33.8 | 48.0 |

**Table B**. **Measurements of signal intensity in key organs and muscle**. CT signal in liver, kidney, spleen, cerebrum, and muscle was measured before and at various time points after intravenous injection of each contrast agent. Data are in HU unit.

| **B1. VivoVist (n=3)** | | | | | | | | | |
| --- | --- | --- | --- | --- | --- | --- | --- | --- | --- |
|  | **Liver** | | | **Kidney** | | | **Spleen** | | |
| Animal | **M1** | **M2** | **M3** | **M1** | **M2** | **M3** | **M1** | **M2** | **M3** |
| Pre-Inj | 40.8 | 44.4 | 39.1 | 19.6 | 23.5 | 13.0 | 44.4 | 52.5 | 32.5 |
| 5min | 186.4 | 196.3 | 186.0 | 127.0 | 111.4 | 118.0 | 196.1 | 237.3 | 217.8 |
| 1hr | 257.4 | 275.2 | 233.4 | 96.7 | 92.3 | 105.9 | 404.8 | 397.0 | 326.7 |
| 4hr | 359.7 | 391.1 | 355.5 | 75.0 | 66.4 | 82.7 | 813.3 | 536.2 | 782.7 |
| 24hr | 385.0 | 407.8 | 412.1 | 26.6 | 35.8 | 34.0 | 1684.5 | 1325.9 | 1537.8 |
| 48hr | 386.9 | 417.3 | 466.9 | 33.3 | 36.7 | 33.5 | 1754.2 | 1430.9 | 1768.6 |
| 96hr | 406.4 | 423.4 | 448.8 | 23.4 | 36.9 | 40.5 | 1561.4 | 1274.2 | 1638.9 |
| 7Days | 400.8 | 436.6 | 484.7 | 28.3 | 37.7 | 36.3 | 1624.4 | 1255.1 | 1499.4 |
|  | **Cerebrum** | | | **Muscle** | | |  | | |
| Animal | **M1** | **M2** | **M3** | **M1** | **M2** | **M3** |  |  |  |
| Pre-Inj | 59.0 | 48.0 | 43.9 | 30.9 | 6.2 | 27.4 |  |  |  |
| 5min | 81.1 | 64.1 | 56.8 | 15.3 | 4.2 | 38.3 |  |  |  |
| 1hr | 67.3 | 58.0 | 57.5 | 23.4 | 17.7 | 27.9 |  |  |  |
| 4hr | 54.8 | 55.3 | 53.2 | 6.9 | 21.2 | 49.5 |  |  |  |
| 24hr | 53.7 | 49.7 | 45.2 | 57.2 | 4.7 | 16.4 |  |  |  |
| 48hr | 59.2 | 54.6 | 46.8 | 25.0 | 2.8 | 29.0 |  |  |  |
| 96hr | 57.0 | 51.8 | 47.8 | 32.9 | 24.0 | 7.3 |  |  |  |
| 7Days | 59.1 | 53.1 | 51.4 | 40.5 | 7.7 | 6.0 |  |  |  |

| **B2. Mvivo-Au (n=4)** | | | | | | | | | | | | | | | | | | | | |
| --- | --- | --- | --- | --- | --- | --- | --- | --- | --- | --- | --- | --- | --- | --- | --- | --- | --- | --- | --- | --- |
|  | **Liver** | | | | | | | **Kidney** | | | | | | **Spleen** | | | | | | |
| Animal | **M4** | | **M5** | **M6** | | **M7** | | **M4** | | **M5** | | **M6** | **M7** | **M4** | | **M5** | | **M6** | | **M7** |
| Pre-Inj | 41.7 | | 26.8 | 33.4 | | 50.3 | | 9.1 | | 5.3 | | 14.1 | 9.1 | 31.5 | | 20.9 | | 42.4 | | 26.7 |
| 5min | 90.9 | | 68.1 | 60.9 | | 88.7 | | 71.8 | | 67.8 | | 78.0 | 61.4 | 108.3 | | 102.0 | | 113.8 | | 85.9 |
| 1hr | NA | | 67.5 | 61.4 | | 82.0 | | NA | | 67.1 | | 81.6 | 49.3 | NA | | 105.1 | | 104.6 | | 90.9 |
| 4hr | 99.3 | | 130.8 | 111.0 | | 99.9 | | 72.7 | | 57.0 | | 83.5 | 51.4 | 106.8 | | 101.2 | | 126.9 | | 82.0 |
| 24hr | 91.9 | | 129.3 | 120.0 | | 89.2 | | 50.2 | | 38.1 | | 59.0 | 36.5 | 107.2 | | 132.4 | | 101.0 | | 86.9 |
| 48hr | 43.3 | | 130.3 | 102.5 | | 96.2 | | 39.5 | | 33.9 | | 44.7 | 24.5 | 131.6 | | 102.7 | | 112.0 | | 88.0 |
| 96hr | 131.9 | | 148.3 | 147.9 | | 101.4 | | 22.3 | | 16.7 | | 22.3 | 19.6 | 190.6 | | 189.1 | | 186.6 | | 111.4 |
| 7Days | 126.6 | | 154.8 | 162.4 | | 120.7 | | 22.6 | | 13.3 | | 30.6 | 18.2 | 204.1 | | 218.3 | | 196.2 | | 112.4 |
|  | **Cerebrum** | | | | | | | **Muscle** | | | | | |  | |  | |  | |  |
| Animal | **M4** | | **M5** | **M6** | | **M7** | | **M4** | | **M5** | | **M6** | **M7** |  | |  | |  | |  |
| Pre-Inj | 38.4 | | 39.3 | 55.5 | | 45.8 | | 39.9 | | 45.3 | | 32.4 | 11.2 |  | |  | |  | |  |
| 5min | 47.8 | | 48.5 | 74.6 | | 57.9 | | 44.2 | | 50.1 | | 30.7 | 10.2 |  | |  | |  | |  |
| 1hr | NA | | 48.1 | 66.9 | | 52.9 | | NA | | 49.6 | | 50.4 | 12.6 |  | |  | |  | |  |
| 4hr | 40.8 | | 38.5 | 69.3 | | 53.1 | | 44.1 | | 35.9 | | 68.3 | 24.7 |  | |  | |  | |  |
| 24hr | 47.8 | | 37.1 | 60.3 | | 49.6 | | 32.6 | | 83.1 | | 12.9 | 13.7 |  | |  | |  | |  |
| 48hr | 40.8 | | 40.8 | 59.9 | | 49.9 | | 49.0 | | 35.7 | | 46.2 | -5.8 |  | |  | |  | |  |
| 96hr | 34.3 | | 45.3 | 55.8 | | 52.4 | | 40.1 | | 40.5 | | 39.0 | 21.4 |  | |  | |  | |  |
| 7Days | 43.0 | | 41.5 | 54.1 | | 53.3 | | 41.7 | | 33.8 | | 56.4 | 1.7 |  | |  | |  | |  |
| **B3. Fenestra-HDVC (n=3)** | | | | | | | | | | | | | | | | | | | | |
|  | | **Liver** | | | | | | | **Kidney** | | | | | | **Spleen** | | | | | |
| Animal | | **M8** | | | **M9** | | **M10** | | **M8** | | **M9** | | **M10** | | **M8** | | **M9** | | **M10** | |
| Pre-Inj | | 39.1 | | | 23.4 | | 54.6 | | 13.5 | | 8.1 | | 54.9 | | 33.1 | | 36.8 | | 67.3 | |
| 5min | | 79.1 | | | 82.4 | | 61.0 | | 60.0 | | 57.8 | | 70.2 | | 78.7 | | 99.3 | | 80.7 | |
| 1hr | | 90.9 | | | 88.8 | | 82.9 | | 64.7 | | 71.8 | | 67.1 | | 112.7 | | 124.0 | | 100.5 | |
| 4hr | | 103.9 | | | 104.4 | | 92.1 | | 62.7 | | 68.2 | | 71.8 | | 119.2 | | 128.8 | | 124.7 | |
| 24hr | | 190.2 | | | 199.9 | | 152.7 | | 26.8 | | 25.4 | | 49.2 | | 142.3 | | 166.8 | | 182.7 | |
| 48hr | | 191.1 | | | 201.2 | | NA | | 26.2 | | 24.5 | | NA | | 174.7 | | 182.7 | | NA | |
| 96hr | | 195.8 | | | 195.0 | | 189.0 | | 20.2 | | 21.8 | | 32.1 | | 173.7 | | 186.7 | | 200.4 | |
| 7Days | | 177.6 | | | 196.9 | | 180.1 | | 17.6 | | 23.0 | | 24.5 | | 168.4 | | 206.5 | | 206.1 | |
|  | | **Cerebrum** | | | | | | | **Muscle** | | | | | |  | | | | | |
| Animal | | **M8** | | | **M9** | | **M10** | | **M8** | | **M9** | | **M10** | |  | |  | |  | |
| Pre-Inj | | 47.8 | | | 44.2 | | 57.7 | | 9.8 | | 33.2 | | 39.5 | |  | |  | |  | |
| 5min | | 56.3 | | | 56.8 | | 35.8 | | 33.7 | | 32.5 | | 27.9 | |  | |  | |  | |
| 1hr | | 51.5 | | | 55.8 | | 35.3 | | 28.1 | | 37.3 | | 19.0 | |  | |  | |  | |
| 4hr | | 53.1 | | | 55.5 | | 31.9 | | 14.0 | | 28.4 | | 18.3 | |  | |  | |  | |
| 24hr | | 44.0 | | | 44.9 | | 34.6 | | 13.2 | | 22.6 | | 27.1 | |  | |  | |  | |
| 48hr | | 47.4 | | | 47.3 | | NA | | 29.7 | | 34.0 | | NA | |  | |  | |  | |
| 96hr | | 50.2 | | | 50.6 | | 44.0 | | 13.7 | | 15.9 | | 19.1 | |  | |  | |  | |
| 7Days | | 50.5 | | | 53.8 | | 40.5 | | 11.8 | | 14.9 | | 16.9 | |  | |  | |  | |
